# Ambulantisierungspotenzial stationärer Fälle einer universitären Klinik für Orthopädie und Unfallchirurgie

**DOI:** 10.1007/s00113-021-01072-w

**Published:** 2021-08-31

**Authors:** Jeanette Henkelmann, Ralf Henkelmann, Nikolaus von Dercks

**Affiliations:** 1 Klinik und Poliklinik für Diagnostische und Interventionelle Radiologie, Universitätsklinikum Leipzig AöR, Liebigstraße 20, 04103 Leipzig, Deutschland; 2 Klinik und Poliklinik für Orthopädie, Unfallchirurgie und Plastische Chirurgie, Universitätsklinikum Leipzig AöR, Leipzig, Deutschland; 3 Stabstelle Medizincontrolling, Universitätsklinikum Leipzig AöR, Leipzig, Deutschland

**Keywords:** Ambulantes Operieren, DRG, Kodierung, MDK-Prüfung, Abrechnung, Outpatient surgery, DRG, Medical coding, Medical audit, Billing

## Abstract

**Hintergrund:**

Durch das Reformgesetz des Medizinischen Dienstes der Kassen (MDK) soll u. a. eine Verlagerung bislang stationär erbrachter Leistungen in den ambulanten Versorgungsektor bzw. die Versorgung nach §115b SGB V umgesetzt werden. Ziel dieser Arbeit ist die Untersuchung bestimmter Gruppen stationärer Fälle eines universitären Maximalversorgers für Unfallchirurgie und Orthopädie, die das Risiko einer operativen Ambulantisierung tragen.

**Methodik:**

Die Datenerfassung mittels SAP Data Warehouse umfasst alle stationären Fälle 2017–2019. Es erfolgt die Subgruppenanalyse der Krankenhausleistungsparameter von 3 potenziellen Risikogruppen (RG): 1) primäre Fehlbelegungen, 2) Katalogleistungen der AOP-Kategorie 1 und/oder 2 sowie 3) elektive Eintagesfälle als hypothetische Risikogruppe. Zudem erfolgt eine Analyse epidemiologischer und ökonomischer Parameter.

**Ergebnisse:**

Eine primäre Fehlbelegung (RG 1) wurde vom MDK in 245 Fällen beanstandet. RG 2 umfasst 764 Fälle und RG 3 891 Fälle. Das Kollektiv wies ein Durchschnittsalter von 45,5 ± 17,7 Jahren auf und zeigte in 90 % keine relevanten Nebendiagnosen (PCCL 0). Der Hauptanteil der Fälle ließ sich den DRG I23B und I21Z (Entfernung von Osteosynthesematerial, 15–23 %) zuordnen, nachfolgend offenen oder arthroskopischen Eingriffen an den Extremitäten (DRG I32F, I32G, I24Z, I18B, 6–9 %). Im Falle einer zunehmenden Ambulantisierung ergibt sich ein potenzielles Erlösrisiko 2017 von 1.049.207 €, 2018 von 1.076.727 € und 2019 von 923.163 €.

**Schlussfolgerung:**

Einzelne Gruppen haben ein erhöhtes Transferpotenzial in bestimmten DRG für ambulante Operationen. Eine proaktive Patientensteuerung in Bezug auf ambulante vs. stationäre Behandlung sowie ein besonderes Management personeller und räumlicher Ressourcen sind notwendig, um nachgelagerte Erlöskürzungen zu antizipieren.

## Abstract (en)

**Background:**

The act of the Medical Service of the Health Funds (MDK) is intended to shift health services previously provided in inpatient care to the outpatient care sector in accordance with § 115b SGB V. The aim of this study was the investigation of potential groups of inpatient cases of a university hospital for trauma surgery and orthopedics, which might be at risk for transfer to surgical outpatient treatment.

**Methods:**

Data collection using SAP Data Warehouse included all inpatient cases 2017–2019, with subgroup analysis of economic parameters of three risk groups (RG): 1) primary misallocation, 2) procedures of AOP categories 1 and/or 2 and 3) elective 1‑day cases. Furthermore, an analysis of epidemiological parameters and an economic evaluation were performed.

**Results:**

Primary misallocations related to 245 cases, RG 2 had 764 cases and RG 3 had 891 cases. The average age was 45.5 ± 17.7 years and in 90% there were no relevant comorbidities (PCCL 0). The majority of cases were assigned to DRG I23B and I21Z (removal of osteosynthesis material, 15–23%), followed by open or arthroscopic surgery of the extremities (DRG I32F, I32G, I24Z, I18B, 6–9%). In cases of a statutory shift of inpatient to outpatient surgical procedures, the potential loss of revenue in 2017 was € 1,049,207, in 2018 € 1,076,727 and in 2019 € 923,163.

**Conclusion:**

Individual groups have an increased potential in certain DRGs for a shift from inpatient to outpatient surgical procedures and are at risk for relevant revenue reductions in the course of further expansion of outpatient care. Proactive patient management in terms of outpatient versus inpatient treatment and special management of staff and spatial resources are necessary to anticipate potential downstream revenue cuts.

Die Rangfolge „ambulant vor stationär“ folgt dem Wirtschaftlichkeitsgebot unseres Gesundheitssystems. Das MDK-Reformgesetz fordert nun eine weitere Verlagerung bislang stationär erbrachter Leistungen in den ambulanten Versorgungsektor nach § 115b SGB V [6]. Durch eine geplante Erweiterung des Katalogs für ambulante Operationen und stationäre Behandlungen soll das Ambulantisierungspotenzial gefördert und gleichzeitig dem Entstehen eines der häufigsten Überprüfungsgründe entgegengewirkt werden.

## Hintergrund und Fragestellung

Der medizinische Fortschritt ermöglicht die Durchführung vieler Operationen ambulant mindestens in gleicher, wenn nicht sogar in überlegener Qualität und zu geringeren Kosten als stationär [1, 2, 2, 15–18, 20, 23]. Vorteile können medizinisch wissenschaftlich verortet sein, z. B. in Form einer geringeren Exposition gegenüber oft problematischen nosokomialen Krankheitserregern. Zudem entspricht die ambulante Durchführung dem Patientenbedürfnis, einhergehend mit einer geringeren psychischen Belastung [3, 10].

Mit dem Gesetz für bessere und unabhängigere Prüfungen des Medizinischen Dienstes der Krankenversicherung (MDK-Reformgesetz) soll u. a. das ambulante Potenzial auf der Grundlage der aktuellen medizinischen Erkenntnisse neu definiert werden [8]. Die Rangfolge „ambulant vor stationär“ folgt dem Wirtschaftlichkeitsgebot gemäß § 12 sowie dem § 39 SGB V [5, 7]. Demnach sollen bestehende ambulante Behandlungsmöglichkeiten in Krankenhäusern durch Erweiterung des Katalogs für ambulante Operationen und stationäre Ersatzverfahren ausgeweitet und gleichzeitig dem Entstehen eines der häufigsten Überprüfungsgründe seitens der Kostenträger entgegengewirkt werden. Nach § 115b Absatz 1a SGB V hätte durch den Spitzenverband Bund der Krankenkassen, die Deutsche Krankenhausgesellschaft und die kassenärztlichen Bundesvereinigungen bis zum 30.06.2020 ein gemeinsames Gutachten beauftragt werden sollen, in dem der Stand der medizinischen Erkenntnisse zu ambulant durchführbaren Operationen, stationsersetzenden Eingriffen und Behandlungen (AOP-Katalog) untersucht wird [8]. Veranlasst durch die COVID-19-Pandemie und auf Grundlage des Zweiten Gesetzes zum Schutz der Bevölkerung bei einer epidemischen Lage von nationaler Tragweite wurden die Implementierung und Anwendung des überarbeiteten AOP-Katalogs auf das Jahr 2022 verschoben [9].

Als Ziel dieser Arbeit soll das Ambulantisierungsrisiko stationärer Fälle der Klinik und Poliklinik für Orthopädie, Unfallchirurgie und Plastische Chirurgie (KOUP) des Universitätsklinikums Leipzig untersucht werden. Für einen Transfer potenziell ambulant behandelbarer Patienten sollen entsprechende Risikogruppen herausgearbeitet und ökonomisch bewertet werden.

## Methodik

### Datenerhebung

Die Datengrundlage umfasst alle entlassenen stationären Fälle der Jahre 2017, 2018 und 2019 der KOUP, welche sich auf die 5 fachlichen Organisationseinheiten Unfallchirurgie (UCh), Arthroskopie/gelenkerhaltende Chirurgie (ACh), plastische und Handchirurgie (PCh), Orthopädie (ORT) und Wirbelsäulenchirurgie (WCh) verteilen. Die Erfassung der Falldaten erfolgte mittels SAP Data Warehouse (Fa. SAP Deutschland, Walldorf) hinsichtlich epidemiologischer Merkmale (Alter, Geschlecht) sowie der Leistungsparameter (Hauptdiagnose (ICD-10-GM), Verweildauer, patientenbezogener klinischer Schweregrad [PCCL]). Der PCCL als kumulativer Schweregrad von Komplikationen und Komorbiditäten der einzelnen Diagnosen wird in den Stufenwerten 0 bis 6 durch den DRG-Grouper ausgegeben.

### Risikogruppen

Aus allen Fällen wurden 3 Risikogruppen gebildet:

- Gruppe 1 (primäre Fehlbelegungen): Hierfür erfolgte die Erfassung der Behandlungsfälle mit primärer Fehlbelegung nach MDK-Prüfung der Jahre 2017–2019. Diese Gruppe hat keine Schnittmenge mit Gruppe 2 und 3, da die Fälle nach der Prüfung nicht mehr als „stationär“ im SAP-Datenbestand geführt werden.
- Gruppe 2 (AOP-Katalog Kategorie 1 und/oder 2): Es wurden die Behandlungsfälle erfasst, deren operativer Eingriff entsprechend dem OPS-Kode ausschließlich einer Kategorie 1 und/oder 2 des AOP-Katalogs zugeordnet werden kann. Fälle mit zusätzlichen Prozeduren ohne Zuordnung zu einer dieser Kategorien wurden ausgeschlossen.
- Gruppe 3 (elektive Eintagesfälle): Hierunter wurden alle Behandlungsfälle mit folgenden Merkmalen erfasst: Alter ≥ 18 Jahre, Verweildauer ein Tag, Beatmungsstunden = 0, DRG-Partition: operativ, PCCL = 0, Aufnahmeart: (normale) Einweisung, Entlassart: (normale) Entlassung.

Die Risikogruppen 2 und 3 entstammen einer gemeinsamen Datengrundlage und weisen eine Schnittmenge auf. Um eine doppelte ökonomische Betrachtung zu vermeiden, wird die Schnittmenge gesondert erfasst.

Die potenziellen Erlöse aus dem ambulanten Operieren wurden als Produkt aus dem mittleren Erlös des ambulanten Operierens des jeweiligen Jahres mit der Fallzahl an Risikofällen des gleichen Jahres ermittelt.

### Datenanalyse

Es werden gängige Größen wie Fallzahl, Case-Mix (CM, Summe der Bewertungsrelationen aller Fälle) und Case-Mix-Index (CMI, Summe der Bewertungsrelationen, dividiert durch die Fallzahl [durchschnittliche Bewertungsrelation pro Fall]), berücksichtigt. Der Landesbasisfallwert (LBFW) beträgt für Sachsen 2017 3278,19 €, 2018 3341,67 € und für 2019 3528,65 €. Es sollen eine epidemiologische und ökonomische Bewertung der Behandlungsfälle der einzelnen Risikogruppen und eine fiktive Erlös- bzw. Verlustrechnung erfolgen. Die Auswertung erfolgte mit Microsoft Excel Version 2013 (Fa. Microsoft Corporation, Redmond, USA) und mittels SPSS Statistics, Version 24.0 (Fa. IBM, Armonk, New York, USA).

## Ergebnisse

Im erfassten Zeitraum von 36 Monaten wurden insgesamt 19.374 Fälle in die Analyse einbezogen, die sich in der Subgruppenanalyse wie folgt darstellen:

### Risikogruppe 1.

Primäre Fehlbelegungen wurden 2017 in 115 Fällen dokumentiert, 2018 in 106 Fällen und 2019 in 24 Fällen. Das mittlere Alter betrug 43,7 ± 19,6 Jahre (62 % weiblich). Der PCCL wies in 92 % der Fälle den Wert 0 auf, (PCCL 1: 5 %, PCCL 2: 3 %). 47 % entstammen der UCh, 27 % der WCh, 11 % der PCh, 8 % der ACh, 7 % der ORT.

### Risikogruppe 2.

Im Jahr 217 erfolgten bei 284 Fällen ausschließlich Prozeduren der Kategorie AOP 1 und/oder 2, in 2018 bei 257 Fällen und in 2019 bei 223 Fällen mit einem mittleren Alter von 44,4 ± 18,8 Jahren (63 % männlich). Der PCCL betrug in 90 % den Wert 0 (PCCL 1: 5 %, PCCL 2: 3 %, PCCL 3: 2 %). Die meisten Fälle entstammen der UCh (51 %), gefolgt von der ACh (22 %), PCh (19 %), ORT (6 %) und WCh (2 %). Eine Operation erfolgte am häufigsten aufgrund der Hauptdiagnosegruppe S83 (Luxation, Verstauchung oder Zerrung des Kniegelenks und von Bändern des Kniegelenks, 11 % der Fälle), nachfolgend aufgrund S82 (Unterschenkelfraktur, 9 %), M23 (Binnenschädigung des Kniegelenks, 6 %) S52 (Fraktur des Unterarms, 6 %) und S42 (Schulter- und Oberarmfraktur, 4 %).

### Risikogruppe 3.

Für die hypothetische Risikogruppe an elektiven Eintagesfällen ergibt sich im Jahr 2017 eine Fallzahl von 281, 2018 von 327 und 2019 von 283 mit einem mittleren Alter von 46,4 ± 16,6 Jahren (53 % männlich). In über 91 % betrug der PCCL den Wert 0 (PCCL 1: 2 %, PCCL 2: 4 % und PCCL 3: 2 %). 44 % der Fälle entstammen der UCh, gefolgt von der ACh (30 %), PCh (15 %), ORT (10 %) und WCh (1 %). Die häufigste Hauptdiagnosegruppe war S52 (18 %), gefolgt von M23 (10 %), S82 (8 %), S42 (6 %) und S83 (4 %).

### Schnittmenge.

Die Risikogruppen 2 und 3 weisen eine Schnittmenge von 98 Fällen bzw. 51,60 CM-Punkten in 2017, 103 Fällen bzw. 54,58 CM-Punkten in 2018 und 73 Fällen bzw. 40,30 CM-Punkten in 2019 auf.

**Figure 1:**
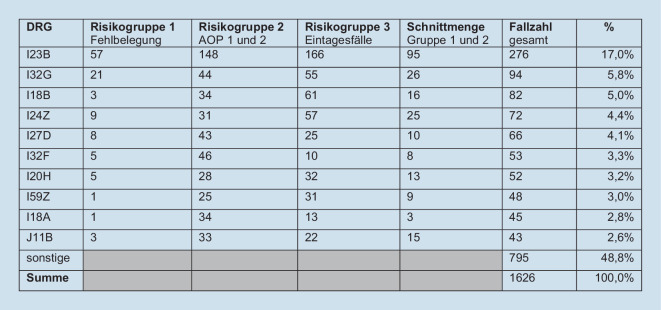

Die Verteilung der DRG nach Risikogruppe zeigt Abb. 1 (exklusive der Schnittmengenfälle). Die 10 häufigsten Fallpauschalen bilden 51,1 % der Risikofälle ab.

### Ökonomische Betrachtung

**Figure 2:**
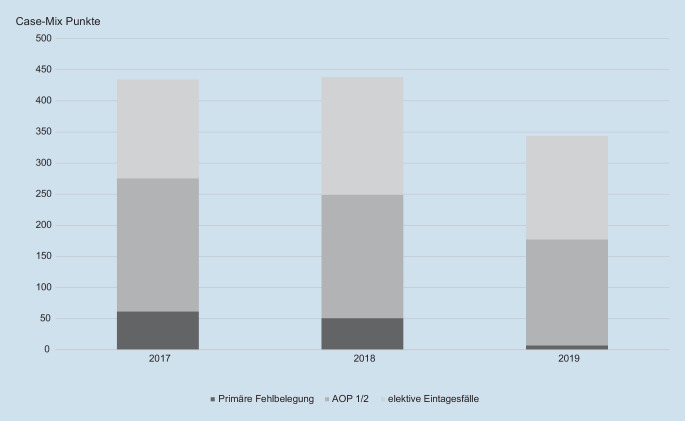

Die Verteilung der CM-Punkte ist in Abb. 2 dargestellt. Die Summe von Fallzahl, CM und Belegtagen pro Jahr ergibt sich durch Addition der jeweiligen Werte abzüglich der Schnittmenge der Risikogruppen 2 und 3. Durch Multiplikation der jeweiligen LBFW ergeben sich aus dem CM die gefährdeten Erlössummen der 3 Jahre. Das ökonomische Risiko der 3 betrachteten Gruppen ist in Tab. 1 zusammengefasst. Den Erlösverlusten aus der Ambulantisierung von operativen Eingriffen stehen die (fiktiven) Erlöse für das ambulante Operieren gegenüber. Diese betrugen für das Jahr 2017 im Mittel und inklusive einer Sachkostenerstattung 394,65 €, für 2018 415,42 € und für 2019 321,30 €. Würden alle Risikofälle ambulant operiert, lägen die ambulanten Erlöse dafür 2017 bei 229.684 €, 2018 bei 243.853 € und 2019 bei 146.836 €. Das verbleibende Erlösrisiko würde dabei für 2017 1.049.207 €, für 2018 1.076.727 € und für 2019 923.163 € betragen.

**Table 1:** 

Entlassungsjahr	2017			2018			2019		
Risikogruppe	Fälle	CM	Belegtage	Fälle	CM	Belegtage	Fälle	CM	Belegtage
1: Primäre Fehlbelegung	115	61,43	187	106	50,72	121	24	7,09	37
2: AOP 1/2	284	214,04	695	257	198,46	673	223	170,37	516
3: elektive Eintagesfälle	281	158,85	281	327	188,96	327	283	166,07	283
./. Schnittmenge Gr. 2/3	98	51,60	98	103	54,58	103	73	40,30	73
Σ	582	382,71	1065	587	383,56	1018	457	303,23	762
Erlösrisiko	1.278.891 €			1.320.580 €			1.070.000 €		
Mittlerer Verlust pro Fall	2197 €			2250 €			2341 €		

## Diskussion

### MDK-Reformgesetz zur Ambulantisierung

Der gesetzgeberische Wille zu wirtschaftlicherem Agieren im Gesundheitswesen soll u. a. durch das MDK-Reformgesetz und die Verlagerung bislang stationär erbrachter Leistungen in den ambulanten Versorgungsektor bzw. die Versorgung nach § 115b SGB V umgesetzt werden [6]. In Deutschland ist die Anzahl ambulant durchgeführter Operationen im Krankenhaus seit dem Jahr 2008 bis 2017 um ca. 12,1 % gestiegen, aber weniger stark als die Anzahl vollstationärer Operationen (um ca. 23,3 %) [12]. Die Frage nach der Notwendigkeit und Dauer von Krankenhausbehandlungen führt immer wieder zu Diskussionen zwischen Leistungserbringern und Kostenträgern. Ursächlich ist grundlegend die Diskussion über die Bindung von Ressourcen, welche an anderer Stelle besser eingesetzt werden könnten.

Die Gesundheitsversorgung soll nach § 12 des SGB V patientenorientiert so gestaltet werden, wie es individuell medizinisch, qualitativ aber auch ökonomisch sinnvoll ist [5]. Leider ist häufig festzustellen, dass das Bestreben der Kostenträger häufig von einer ökonomischen Maxime geprägt ist. Für Krankenhäuser gab es bislang wenige Anreize für eine Verlagerung stationärer Operationen in die ambulante Versorgung, nicht zuletzt, weil die stationäre Versorgung gegenüber der ambulanten deutlich besser vergütet ist. Weiterhin fehlen am Krankenhaus häufig effiziente Parallelstrukturen für das ambulante Operieren.

### Ambulantisierungsrisiko stationärer Fälle

Ziel dieser Arbeit ist die Bewertung des Ambulantisierungsrisikos stationärer Fälle eines universitären Maximalversorgers für Orthopädie und Unfallchirurgie anhand von 3 Patientengruppen. Bei Gruppe 1 ist das Risiko bereits eingetreten, da diese Fälle nach MDK-Prüfung und Leistungsentscheidung der Krankenkassen bereits einen sog. Fallartwechsel von stationär nach ambulant erfuhren. Aus der Fallübersicht geht hervor, dass häufig DRG der konservativen Wirbelsäulenbehandlung betroffen sind (I68D und I68E, 25 %). Hierbei ist nicht immer von einem realen Transferpotenzial auszugehen, da diese Patienten häufig in akuten Schmerzsituationen eine stationäre Behandlung benötigen. Diesen Patienten ist einem Fallartwechsel durch gewissenhafte Dokumentation zu begegnen. Allerdings fallen auch Patienten, die zu diagnostischen Zwecken bei Wirbelsäulenerkrankungen stationär behandelt werden (z. B. Stufendiagnostik) in die Basis-DRG I68. Insbesondere hier gilt es, die Möglichkeit ambulanter Leistungserbringung zu prüfen.

Auch bei Behandlungsfällen der DRG I23B (Entfernung von Osteosynthesematerial) wird häufig keine stationäre Behandlungsnotwendigkeit gesehen. Diese sollte in der Tat individuell abgewogen werden, und ein Fallartwechsel ist wiederum nur mit einer entsprechenden Dokumentation und Begründung zu verhindern. Selbstredend sei an dieser Stelle auf wahrheitsgemäße Dokumentation hingewiesen, die aber vollständig sein muss, will man damit bei der MD-Begutachtung oder vor dem Sozialgericht Bestand haben. Jedoch zeigen bereits mehrere Studien, dass bereits die initiale operative Versorgung in einem ambulanten Setting durchgeführt werden kann [17, 20].

Gruppe 2 berücksichtigt Fälle, deren stationäre Operationen einem Eingriff der AOP-Kategorie 1/2 entsprechen. Wiederum stellt die DRG I23B die größte Untergruppe dar. Die zugehörigen Hauptdiagnosen sind heterogen, aber größtenteils aus dem Formenkreis von Verletzungen der unteren Extremität (ca. 26 %). Hierbei ist zu berücksichtigen, dass bei Materialentfernungen der Frakturkode die Hauptdiagnose darstellt. Das Ambulantisierungsrisiko ist erneut individuell hoch einzuordnen.

Die Auswahl der Parameter der Gruppe 3 soll den komplikationslosen Eintagesfall beschreiben, deren Charakterisierung durch eine elektive Aufnahme andere Eintagesfälle wie z. B. zur Überwachung nach Schädel-Hirn-Trauma abgrenzt. Die Entlassart „normal“ grenzt Verstorbene ab. Zu den häufigsten Hauptdiagnosen zählen Frakturen des Unterarms, größtenteils distale Radiusfrakturen. Sofern keine offenen Frakturen, große Weichteilschäden oder sonstige komplizierende Umstände vorliegen, erfolgt die Versorgung größtenteils nicht akut [11]. Whiting et al. konnten zeigen, dass eine ambulante Operation geschlossener Frakturen der stationären Behandlung überlegen ist [23]. Hier besteht ein hohes Ambulantisierungsrisiko. Neben der Entfernung von Osteosynthesematerial (I23B) weisen zudem DRG mit Bezug zu arthroskopischen Eingriffen am Kniegelenk (I24Z) ein Ambulantisierungsrisiko auf. Schwappach et al. zeigten in ihrer Patientenbefragung, dass ambulante Operationen einem Patientenbedürfnis entsprechen [21]. Grundlegend ist eine arthroskopische Behandlung von Kreuzbandrupturen und Meniskusnähten bzw. -resektionen ambulant in gleicher Qualität möglich [16, 18, 19, 22].

Die meisten Fälle mit Transferpotenzial können den Organisationseinheiten UCh, ACh und PCh zugeordnet werden. Dabei stellt sich in allen 3 Gruppen am häufigsten die DRG I23B heraus (ca. 19–23 %). In der Regel handelt es sich dabei um elektive stationäre Aufenthalte für Operationen überwiegend an „kleinen Knochen“ wie Schlüsselbein, Unterarm oder am Sprunggelenk, da die aufwendigeren Materialentfernungen (Wirbelsäule und Oberschenkel) nicht in dieser DRG enthalten sind. Prinzipiell kann hier eine ambulante Versorgung erwogen werden. Kritisch ist jedoch anzumerken, dass es sich auch um Ausbildungsinhalte handelt und ein komplettes Streichen dieser Operationen aus dem stationären Portfolio die Ausbildung schwächt. Daher wäre entweder ein Teiltransfer oder die Einbindung von Ausbildungsassistenten in das ambulante Setting zu erwägen.

Insgesamt zeichnen sich die Gruppen durch ein junges Patientenkollektiv aus (knapp über 40 Jahre), welches in ca. 90 % keine relevanten Nebendiagnosen (PCCL 0) aufweist und potenziell ambulantisierbar erscheint. Aus dem „Katalog ambulant durchführbarer Operationen und sonstiger stationsersetzender Eingriffe“ kann natürlich nicht die Verpflichtung hergeleitet werden, die dort aufgeführten Eingriffe ausschließlich ambulant zu erbringen. Grundsätzlich entscheidet der verantwortliche Arzt individuell über Art, Umfang und Setting des operativen Eingriffs mit Hinblick auf den Gesundheitszustand und eine angemessene Nachbetreuung im häuslichen Bereich. Als Begründungs- und Bewertungshilfe haben die Selbstverwaltungspartner dazu den sog. G‑AEP-Katalog abgestimmt (German Appropriate Evaluation Protocol). Eine verpflichtende Grundlage für oder gegen eine stationäre Behandlung stellt dieser jedoch nicht dar, sondern gibt vielmehr lediglich Orientierungskriterien.

Von den 3 Gruppen spiegelt Gruppe 1 das bereits eingetretene Risiko der Ambulantisierung wider. Das maximale Risiko stellt den Wegfall der Erlöse aller 3 Gruppen dar. Demgegenüber sind die ambulanten Erlöse gering und fangen den potenziellen Erlösausfall in keinem Fall adäquat ab. Bezogen auf die untersuchte Klinik bedeutet der potenzielle Erlösverlust rund 3–4 % der DRG-Erlöse des jeweiligen Jahres.

### Perspektive ambulantisierbarer Operationen

Typischerweise ist eine Unfallchirurgie der Maximalversorgung für die Behandlung von Schwerstverletzten zuständig und strukturell und personell dahingehend organisiert. Durch die Inanspruchnahme stationärer Kapazitäten für dringlichere Eingriffe entstehen mitunter unverhältnismäßig lange Wartezeiten für elektiverer Operationen. Eine zunehmende Integration ambulanter Operationen mit einer entsprechenden Erwartungshaltung ist eine große Herausforderung. Demgegenüber hat die synergistische und effiziente Nutzung bestehender Ressourcen auch für ambulante Operationseinheiten Potenzial und ist als vorteilhaft anzusehen. Nicht nur strukturelle Ressourcen wie z. B. Apotheken- oder Sterilisationseinheiten, sondern auch persönliche Fähigkeiten wie eine entsprechende Qualifizierung von Fach- und Pflegepersonal können integriert werden. Die Vermischung dieser Ressourcen ist wiederum komplex, und nicht selten entstehen hierbei Interessenkonflikte.

Als ökonomischer Anreiz einer zunehmenden Ambulantisierung operativer Eingriffe wird der Ausschluss ambulanter Leistungen des AOP-Katalogs von MD-Prüfungen gesehen. Hinzu kommt die eingeführte Strafzahlung für jede beanstandete Krankenhausrechnung ab 2022 (mind. 300 € bzw. höchstens 10 % des durch den MD geminderten Abrechnungsbetrags) [4]. Die Steigerung ambulanter Eingriffe könnte hier Streitpotenzial mindern. Ein nicht von der Hand zu weisender Vorteil des ambulanten Operierens ist die direkte Sachkostenerstattung außerhalb des Sprechstunden- und Praxisbedarfs und unter Einhaltung des Wirtschaftlichkeitsgebots, die in § 44 des Bundesmantelvertrags-Ärzte (BMV-Ä) geregelt ist [14].

Generell sind für einen praktischen Falltransfer stationär nach ambulant diverse Herausforderungen personeller, räumlicher und struktureller Art zu berücksichtigen. Ein funktionierendes Netzwerk, Kooperationen und ein sicher geplantes Management sind uneingeschränkte Voraussetzungen für eine funktionierende Integration des ambulanten Operierens in ein Krankenhaus. Eine Umsetzung der Fallsteuerung ist ebenso durch die Anbindung eines MVZ und ambulantes Operationszentrums möglich und wird zunehmend in Deutschland umgesetzt [13]. Die Integration und Überschneidung stationärer und ambulanter Prozesse wird effektiven Nutzen durch synergistische Ressourcennutzung bringen, wenn die Hürden eines komplexen Managements überwunden werden können.

Limitationen: Methodenkritisch ist zu erwähnen, dass es sich bei der vorliegenden Arbeit um eine „Single-center“-Studie an einem Universitätsklinikum handelt. Das zugrunde liegende Patientenkollektiv und die daraus abgeleiteten Risikopotenziale sind daher nicht ohne Weiteres auf andere Krankenhäuser zu übertragen. Das Rechnungsjahr 2019 ist mit den vorliegenden Zahlen noch nicht vollständig bezüglich der MD-Gutachten bereinigt. Die Fallzahl der primären Fehlbelegungen liegt voraussichtlich in der Größenordnung der Vorjahre.

## Fazit für die Praxis

- Mit dem MDK-Reformgesetz soll das Ambulantisierungspotenzial weiter gefördert werden.
- Vor allem elektive „kleinchirurgische“ Eingriffe wie die Entfernung von Osteosynthesematerial bieten ein Transferpotenzial zum ambulanten Eingriff.
- Kritisch anzumerken ist, dass es sich hier aber auch um Ausbildungsinhalte handelt. Zudem sind für einen praktischen Falltransfer stationär nach ambulant diverse Herausforderungen personeller, räumlicher und struktureller Art zu berücksichtigen.
- In jedem Fall muss vorab eine entsprechende Vorselektion des Patientenguts erfolgen.
